# Identification of miRNAs and their target genes in *Larix olgensis* and verified of differential expression miRNAs

**DOI:** 10.1186/s12870-019-1853-4

**Published:** 2019-06-11

**Authors:** Sufang Zhang, Shanshan Yan, Jiali Zhao, Huanhuan Xiong, Peiqi An, Junhui Wang, Hanguo Zhang, Lei Zhang

**Affiliations:** 1 State Key Laboratory of Tree Genetics and Breeding (Northeast Forestry University), Harbin, 150040 China; 2 State Key Laboratory of Tree Genetics and Breeding (Chinese Academy Of Forestry), Beijing, 100081 China

**Keywords:** miRNA, *Larix olgensis*, Target gene, Differential expression

## Abstract

**Background:**

MiRNAs (microRNA) are 18–24 nt endogenous noncoding RNAs that regulate gene expression at the post-transcriptional level, including tissue-specific, developmental timing and evolutionary conservation gene expression.

**Results:**

This study used high-throughput sequencing technology for the first time in *Larix olgensis*, predicted 78 miRNAs, including 12,229,003 reads sRNA, screened differentially expressed miRNAs. Predicting target genes was helpful for understanding the miRNA regulation function and obtained 333 corresponding target genes. Gene Ontology (GO) and Kyoto Encyclopedia of Genes and Genomes (KEGG) functional annotation were analysed, mostly including nucleic acid binding, plant hormone signal transduction, pantothenate and CoA biosynthesis, and cellulose synthase. This study will lay the foundation for clarifying the complex miRNA-mediated regulatory network for growth and development. In view of this, spatio-temporal expression of miR396, miR950, miR164, miR166 and miR160 were analysed in *Larix olgensis* during the growth stages of not lignified, beginning of lignification, and completely lignified in different tissues (root, stem, and leaf) by quantitative real-time PCR (qRT-PCR). There were differences in the expression of miRNAs in roots, stems and leaves in the same growth period. At 60 days, miR160, miR166 and miR396–2 exhibited the highest expression in leaves. At 120 days, most miRNAs in roots and stems decreased significantly. At 180 days, miRNAs were abundantly expressed in roots and stems. Meanwhile, analysis of the expression of miRNAs in leaves revealed that miR396–2 was reduced as time went on, whereas other miRNAs increased initially and then decreased. On the other hand, in the stems, miR166–1 was increase, whereas other miRNAs, especially miR160, miR164, miR396 and miR950–1, first decreased and then increased. Similarly, in the roots, miR950–2 first decreased and then increased, whereas other miRNAs exhibited a trend of continuous increase.

**Conclusions:**

The present investigation included rapid isolation and identification of miRNAs in *Larix olgensis* through construction of a sRNA library using Solexa and predicted 78 novel miRNAs, which showed differential expression levels in different tissues and stages. These results provided a theoretical basis for further revealing the genetic regulation mechanism of miRNA in the growth and development of conifers and the verification of function in target genes.

## Background

MiRNAs are a class of endogenous single-stranded non-coding RNAs approximately 18–24 nt in length. Derived from single-stranded precursor RNAs that can form hairpin structures [1, 2], miRNAs regulate gene expression primarily at the post-transcriptional level [3]. As miRNAs are structurally conserved, bioinformatics methods can be used to predict miRNAs and their target genes. Given that miRNA sequences are short and expressed only in specific tissues/cells or at specific stages in cells, researchers have overlooked miRNAs. In 1993, Lee discovered the first miRNA in *Caenorhabditis elegans*(*C. elegans*), miRNA-lin4* [4], which temporally regulates embryonic development. Later, numerous follow-up studies found that in higher plants, miRNA plays an important role at the post-transcriptional level by regulating its target mRNA through silencing or degrading mRNA [5–7]. The first miRNA in plants was identified in *Arabidopsis* in 2002 [8].

Gymnosperms and angiosperms differentiated approximately 300 million years ago. Gymnosperms have evolved and developed distinct miRNAs over a long evolutionary time scale. These distinct miRNAs may play important roles in the growth and development of gymnosperms. As of 2012, miRBase (18.0), a miRNA database established by the University of Manchester and the University of Cambridge, has published 18,226 miRNA precursor sequences in 168 species, among which 52 are plant species. However, the majority of plant species are angiosperms, and *Pinus taeda* and *Picea asperata* represent the only gymnosperms included in the database. The scarcity of miRNA information from gymnosperms has greatly hindered the progress of miRNA studies. The application of emerging sRNA library high-throughput sequencing technology can help solve this problem, especially for conifers with large genomes and little information on ESTs. Solexa sequencing can obtain miRNA information in a high-throughput, rapid and efficient manner.

miRNA degrades its mRNA target or blocks the translation of the mRNA target mainly by complementary pairing to the mRNA target. When miRNA complementarily pairs with its mRNA target, the bases at positions 2–8 at the 5′ end of the miRNA first specifically pair with the fragment of the target site on the mRNA [9, 10] to form a primary transcript pri-miRNA by RNA polymerase II. Then, mediated by the Dicer-like 1(DCL1) enzyme complex in the nucleus, pri-miRNA is cleaved to form a pre-miRNA with a hairpin structure. Next, pre-miRNA is processed to form a mature miRNA:miRNA* [11, 12]. Finally, one strand of the miRNA:miRNA* binds with the Argonaute1 (AGO1) protein to form an RNA-induced silencing complex (RISC) that performs its function; this strand is termed miRNA and the other complementary strand is miRNA*. Numerous studies have confirmed that miRNA* is readily degraded and may play a very important role as a negative regulator of gene expression in eukaryotes, which to a certain extent broadens the network of miRNA-mediated gene regulation [13]. In plants, a large proportion of miRNAs and their mRNA target genes have less than four mismatches [14]; thus, plant miRNAs perform their functions mainly through binding to the AGO1 protein to form RISC, cleaving from the middle of the pairing region to the 5 ‘end of mRNA [15, 16]. Numerous previous studies have confirmed that most of the target genes of plant miRNAs are transcription factors, stress response factors, and F-box proteins [17–19], underscoring the central position of miRNAs in the expression and regulation of genes involved in plant development. In addition, studies in recent years have found that plant miRNAs are also involved in signal transduction pathways, plant hormone induction, synthesis and metabolism of organic compounds, and ion transport, indicating the important biological significance of miRNAs.

*Larix olgensis* is an important economic and ecological species and a major afforestation species in north-eastern China. The genetic gain of *Larix olgensis* obtained by conventional improvement methods will decrease with continued generations. On the other hand, *Larix olgensis* has a very complex genetic background and a large genome, and studies of the molecular mechanisms of its growth and development lag behind. Therefore, a small RNA library library was constructed using Solexa high-throughput sequencing technology, which allows the rapid isolation and identification of miRNAs. Upon removing low-quality sequences and screening for clean reads, miRNA prediction was followed by gene identification and functional annotation of target genes. Finally, differential expression analysis of miRNAs was performed in *Larix olgensis* by qRT-PCR to gain a preliminary understanding of the regulatory roles of miRNA in the growth and wood property development of *Larix olgensis*.

## Results

### Quality analysis of sRNA library

After testing, the concentration and purity of RNA conformed to the requirements of high-throughput sequencing. Finally, a total of 21,095,170 raw reads for the S01 sample and 16,395,342 raw reads for the S02 samples were obtained to construct the miRNA library. A total of 27.21 M clean reads was obtained. There were 15,294,797 (72.50%) and 11,913,982 (72.67%) clean reads in the S01 and S02 samples, respectively. The proportion of the clean reads in the corresponding raw reads was more than 50% in the two samples, which suggested that the quality of the sequencing data was high.

### Sequence analysis of small RNA

Using Bowtie [20] software, the filtered clean reads were applied to the Silva database, GtRNAdb database, Rfam database and Repbase database for sequence alignment and to filter out ncRNAs, such as rRNA, tRNA, snRNA, snoRNA and repbase sequences to obtain unannotated reads. The classification and annotation of samples S01 and S02 are shown in Table 1.

**Table 1:** sRNA annotation classification statistics of samples

Sample Types	rRNA	scRNA	snRNA	snoRNA	tRNA	Repbase	Unannotated	Total
S01	2,991,972	0	1670	467	65,264	6421	12,229,003	15,294,797
S02	1,951,303	0	1059	322	35,933	4279	9,921,086	11,913,982

Notes: *rRNA* Annotated ribosomal RNA reads, *scRNA* Annotated cytoplasmic small RNA reads, *snRNA* Annotated small RNA reads in the nucleus, *snoRNA* Annotated nucleolide small RNA reads, *tRNA* Annotated the transfer of RNA reads, *Repbase* Annotated repeat sequence reads, *Unannotated* Unannotated reads, they will be used for subsequent analysis, *Total* Filtered reads

### Analysis of miRNAs

Due to the differences in the length percentage of miRNA from different species, the classification statistics for potential miRNA were predicted with length. The 21 nt product was the highest; in the S01 sample, it accounted for 25.76% of all the sequences and in the S02 sample it was 26.71%. The second most common were 20 nt (S01 17.34%, S02 21.11%) and 22 nt (S01 17.11%, S02 16.58%).

**Fig. 1:**
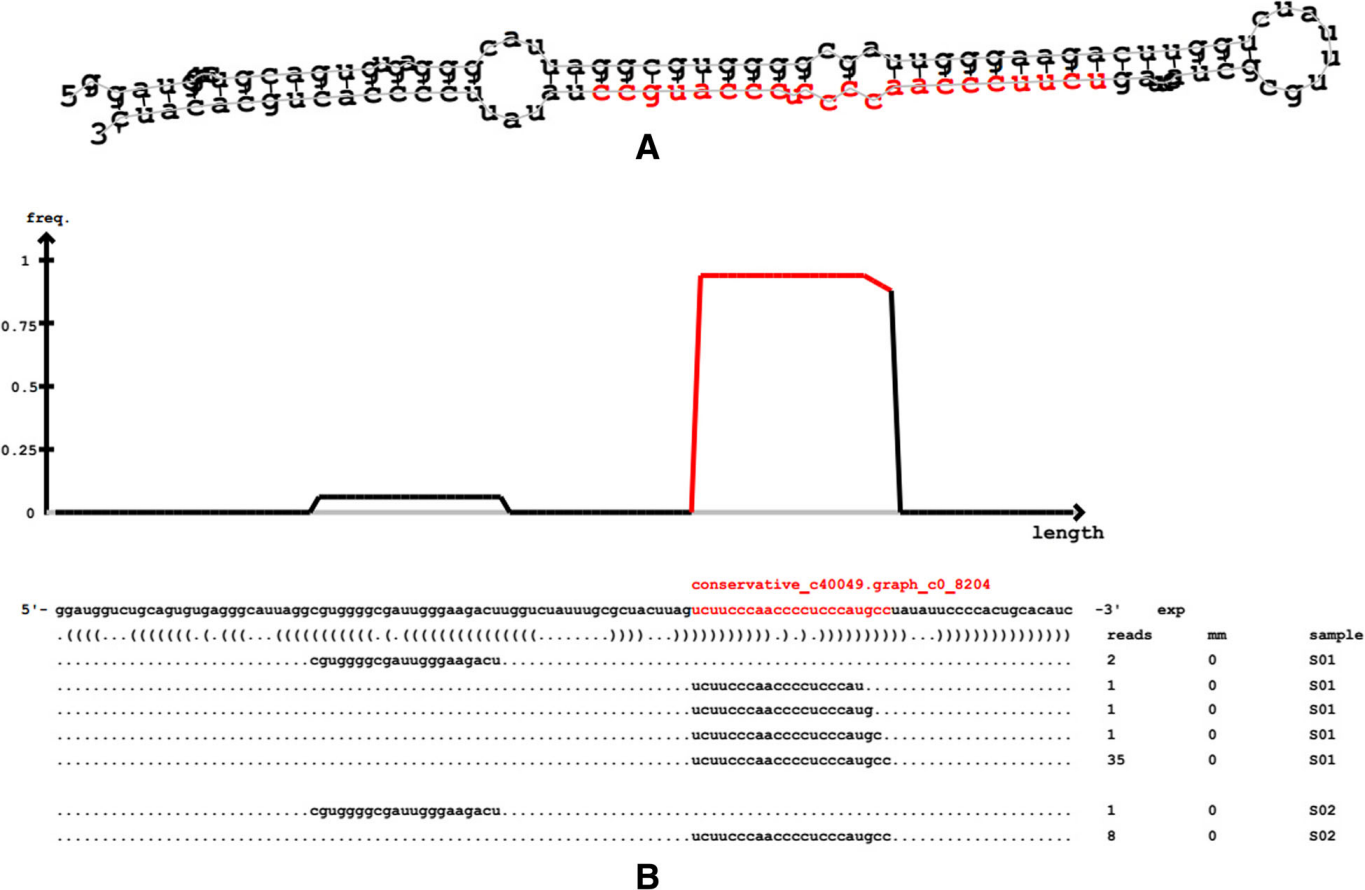
Structure of the miRNA precursor. Notes: Figure A shows the secondary structure prediction of miRNA precursors by miRDeep2 software; The red mark is mature sequence, the right most circle is a ring structure; The above of Figure B is the reads distribution aligned to the precursor of this strip, and the bottom is the location containing the mature sequence

The predicted structure of the miRNA precursor is presented in Fig. 1. A total of 78 new miRNAs was predicted in all samples. Given that no miRNAs in larch were included in the miRBase library, the predicted number of known miRNAs was zero, and all of the predicted miRNAs may be novel miRNAs, including conserved and unconserved. Due to the specificity of Dicer and the DCL enzyme, the lengths of the final mature miRNAs mainly range from 20 to 24 nt. In particular, plant miRNAs were predominantly 21 nt. This result was mainly related to the selected DCL enzyme [21].

The length distribution of the novel miRNAs was studied. The mature miRNA length of *Larix olgensis* was mostly 21 nt, occupying 55.13% in all of the predicted miRNAs, and the predicted miRNA length was mainly concentrated in the range of 21 nt to 22 nt, which was consistent with the conventional conclusion. In addition, studying the base preference in *Larix olgensis* revealed that U at the first base in the 21 nt product accounted for 53.49%, and in 22 nt it was 71.43%, which was higher than the other bases in the position sum of all proportion, the same as the miRDeep2 prediction results.

### Analysis of differentially expressed miRNAs

#### Screening of differentially expressed miRNAs

A total of 49 differentially expressed miRNAs was obtained by comparing the miRNAs among the samples, including 11 conserved miRNAs and 38 unconserved miRNAs. Thirty-three miRNAs were upregulated, and 16 miRNAs were downregulated. Given that miRNAs are involved in various plant life activities and processes and two samples were obtained from the beginning and the peak of the thickening growth period, the growth and development between the two sample time points may be orchestrated by a variety of miRNAs that play important roles in plant growth and development.

#### Cluster analysis of differentially expressed miRNAs

Before screening for differentially expressed miRNAs, the expression levels of miRNAs in both samples were statistically quantified and then normalized using the transcripts per million (TPM) algorithm [22]. The TPM normalization formula is TPM = Readcount*1000/MappedReads, where readcount denotes the number of reads aligned to a given miRNA, and MappedReads denotes the number of reads aligned to the reference genome. The expression abundance was then used to screen for differentially expressed miRNAs.

In the detection of differentially expressed miRNAs for *Larix olgensis* species with no biological replications, differential expression analysis was performed using IDEG6 [23] and employing |log2(FC)| > =1 and FDR < =0.05 as screening criteria, where the fold change (FC) indicates the ratio of the expression level between two samples. Given that the differential expression analysis of miRNAs includes a large number of independent statistical hypothesis tests for miRNA expression levels, a false positive problem exists. To address this problem, the Benjamini-Hochberg correction method [24] was used to adjust the significant *p*-values obtained from the original hypothesis tests, and the false discovery rate (FDR) was used as a key indicator to screen for differentially expressed miRNAs.

**Fig. 2:**
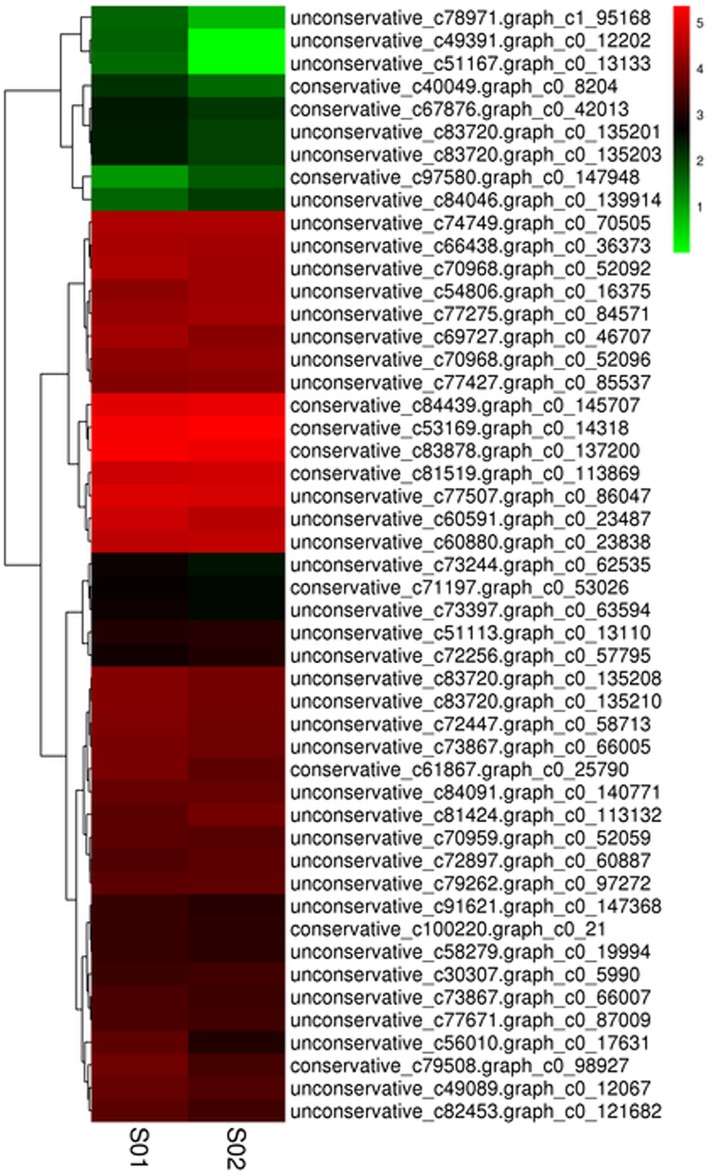
Differentially expressed miRNAs cluster. Notes: columns are different samples; rows are different miRNAs. Clustering with log10 (TPM + 1) value, red is high expressed miRNA, green is low high expressed miRNA

The differentially expressed miRNA was analysed by hierarchical cluster analysis based on complete linkage. The miRNA with the same or similar expression was clustered. The cluster results for differentially expressed miRNAs are shown in Fig. 2. According to the expression abundance, all miRNAs predicted conventional (low expression) and high abundance (high expression) expression.

The differential expression of the same miRNAs in two samples showed that the differentially expressed miRNAs (49) accounted for 62.82% of all the miRNAs (78), and most of the differentially expressed miRNAs were expressed in high abundance. Most of the differentially expressed miRNAs were upregulated. The results showed that the proportion of miRNAs upregulated in sample S01 was higher, while the proportion of miRNAs downregulated in sample S02 was higher. In addition, it can be seen from the figure that some differentially expressed miRNAs were stably expressed, and some were constantly changing with growth and development.

### MiRNA target gene prediction and function annotation

#### Target gene prediction

Plants can exercise their regulatory function by combining miRNA and mRNA or degrading mRNA. Based on the mechanism of plant miRNA, the predicted miRNA and corresponding gene sequence are used with TargetFinder software to predict target genes (Table 2).

**Table 2:** Statistics for miRNA target genes number

Types	All miRNA	miRNA with Target	Target gene
S01	76	76	325
S02	73	73	302
Total	78	78	333

Notes: *Types* miRNA type, *All miRNA* Total number of miRNAs, *miRNA with Target* Number of miRNAs predicted to target genes, *Target Gene* Number of target genes predicted

In the S01 and S02 samples, 76 and 73 miRNAs, respectively, that have corresponding target genes were predicted, and the total numbers of the target genes were 325 and 302, respectively. In the two samples, the total number of target genes for the 78 predicted miRNAs was 333. Differentially expressed miRNAs carry out their functions ultimately through regulating mRNAs. Therefore, to understand the functions and regulation patterns of miRNAs, preliminary understanding and knowledge of their target genes is essential. The results show that one miRNA may regulate more than one target gene. Similarly, a target gene may be regulated by multiple miRNAs.

#### The target gene annotation of differentially expressed miRNAs

The predicted target gene sequences were aligned to NR [25], Swiss-Prot [26], GO [27], COG [28], KEGG [29], KOG [30] and Pfam [31] databases by BLAST software, and the annotation information for target genes was obtained. Annotation information was obtained for only 222 of 333 predicted target genes, and all the miRNA target gene annotation numbers (Table 3) and differential miRNA target gene annotation numbers were counted (Table 4).

**Table 3:** Statistics for the target genes number of all miRNAs

Anno_Database	Annotated_Number	300 < =length < 1000	length > =1000
COG_Annotation	43	4	39
GO_Annotation	140	54	86
KEGG_Annotation	33	4	29
KOG_Annotation	81	15	66
Pfam_Annotation	145	33	112
Swissprot_Annotation	162	52	110
nr_Annotation	220	81	139
All_Annotated	222	82	140

**Table 4:** Statistics for the target gene numbers of differentially expressed miRNAs in two samples

Type	COG	GO	KEGG	Swissprot	NR	KOG	Pfam	Total
S01_vs_S02	12	90	10	92	134	25	79	134

Notes: The first column represents the sample combination, the second column to the last column represents the function database, and the last column represents the sum of the comments

When the target gene sequence length is greater than 1000, the number of target genes that has been annotated through alignment in each database is greater than when the target gene sequence length is between 300 and 1000. Additionally, the total number of annotated target genes for those with sequence length greater than 1000 accounted for 63.06% of the total number of target genes in these databases. Annotation analysis for the target genes of the differentially expressed miRNAs demonstrated that the number of annotated target genes was the largest in the NR database, including all the annotation information for the target genes of differentially expressed miRNAs. The next largest databases were Swiss-Prot and GO, which accounted for 68.66 and 67.16% of the annotation information for the target genes of differentially expressed miRNAs, respectively. The COG and KEGG databases contained the least annotation information. These results indicate that a miRNA target gene has the same miRNAs, whereas a miRNA does not necessarily have a single target gene, which also demonstrates the complexity of miRNAs and their target gene regulatory network.

#### GO annotation for differentially expressed miRNA target genes

**Fig. 3:**
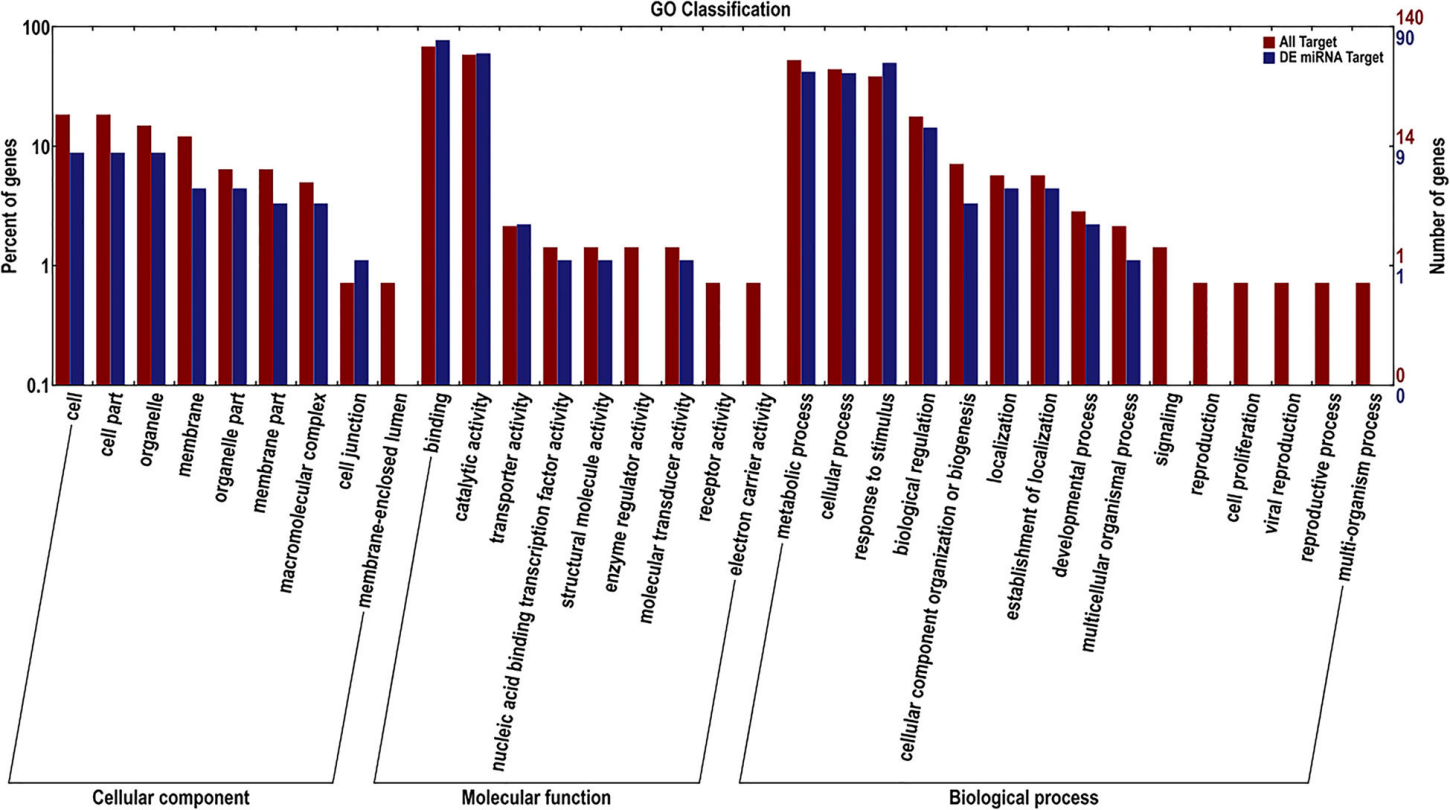
Statistics of GO annotation classification for the target genes of differentially expressed miRNAs. Notes: the abscissa is the GO classification; the ordinate is the percentage of gene number (left) and number of genes (right). This figure shows the gene enrichment of the secondary functions of GO in the background of the target gene and all genes of the differential expression miRNAs, reflecting the status of each secondary function in the two contexts

The GO database is a structured standard biological annotation system applicable to all species, which establishes a standard information system for gene and protein research, and can describe the properties of genes and their products in detail. GO function analysis includes three ontologies: cellular component, molecular function, and biological process [32]. GO classification annotation of target genes predicted for all miRNAs and difference expression miRNAs were analysed and listed as class I and class II, which could reflect biological characteristics of differentially expressed genes. The results are shown in Fig. 3.

GO analysis showed that 563 target genes were annotated in all of the predicted miRNAs. The target gene annotation of difference miRNA was 315, accounting for 55.95% of the total number of target genes annotated. Among them, target gene relevant cellular components were 117, of which 39 were differentially expressed miRNA target genes, accounting for 33.33% of all target genes. The three largest percentages were cell (8), organelle (8) and cell part (8). Target gene relevant molecular functions were 191, of which 129 were differentially expressed miRNA target genes, accounting for 67.54% of all target genes. The catalytic activity (54) term and binding (70) term occupied the largest numbers of genes. Target gene relevant biological processes were 255, of which 147 were differentially expressed miRNA target genes, accounting for 57.65% of all target genes. The three largest percentages were metabolic process (38), cellular process (37) and response to stimulus (45). The figure shows GO secondary function enrichment of all the miRNAs and different expression of target genes, which reflected their positions. Having an obvious proportional difference secondary function suggested that the enrichment tendency was different between differentially expressed miRNA target genes and all target genes. According to enrichment tendency, the life activities direction regulated by miRNAs could be simply predicted, for example, among cellular components, different expression of target genes without membrane-enclosed lumen; among molecular functions, without receptor activity, electron carrier activity, enzyme regulator activity; and among biological processes, without reproduction, viral reproduction, reproductive process, signaling and multi-organism process, suggested that the miRNAs involved in these processes were relatively stably expressed during growth and development in *Larix olgensis*. However, there were also some miRNA target genes that were annotated more than others, such as catalytic activity, binding, response to stimulus, cellular process, and biological regulation, which indicated that more than one miRNA regulated their target genes to complete the normal growth and development process. In conclusion, it can be very intuitive to understand that these GO terms are enriched by different expression of miRNAs in the categories of cellular component, molecular function, or biological processs.

#### COG annotation for differentially expressed miRNA target genes

**Fig. 4:**
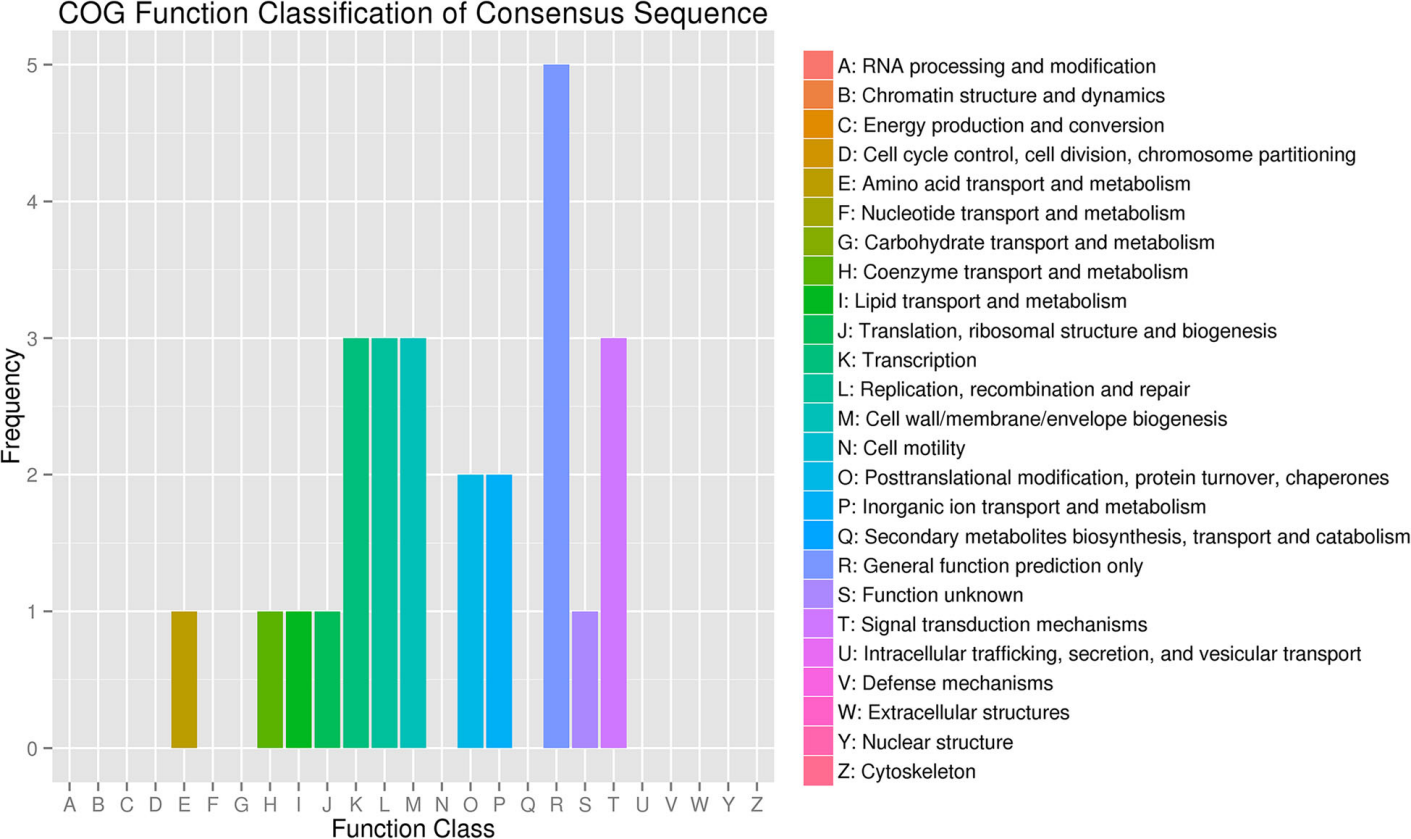
Statistics of COG annotation classification for the target genes of differentially expressed miRNAs. Notes: the abscissa is the COG classification and the ordinate is gene number frequency

The COG database is constructed based on the phylogenetic relationships of bacteria, algae and eukaryotes. The COG database can be used for homologous classification of proteins. COG classification annotation results for differentially expressed miRNA target genes are shown in Fig. 4. These target genes are involved in amino acid transport and metabolism, coenzyme transport and metabolism, lipid, inorganic ion transport and metabolism; translation, ribosomal structure and biogenesis, cell wall/membrane envelope biogenesis; transcription; replication, recombination and repair; life activity processes, such as posttranslational modification, protein turnover, and chaperones; function prediction, such as signal transduction mechanisms. Among the different function classifications, the proportion of differential miRNA target genes annotated reflects the information on the physiological or metabolic bias in the corresponding period and environment. As shown in the figure, relating to these processes were transcription; replication, recombination and repair; cell wall/membrane envelope biogenesis; posttranslational modification, protein turnover, chaperone life activity processes; and coenzyme transport and metabolism. The expression level of these target genes was relatively high, suggesting that the samples were in a vigorous growth and development period. Correspondingly, sample 1 was in the early stages of growth, and sample 2 was in the vigorous growth period. Most miRNAs target genes were in the category of general function prediction and function unknown that the expression level was the highest, suggesting most miRNAs participation in the entire life process and needing further verification.

### Enrichment for differentially expressed miRNAs

#### GO enrichment for the target genes of differentially expressed miRNAs

**Fig. 5:**
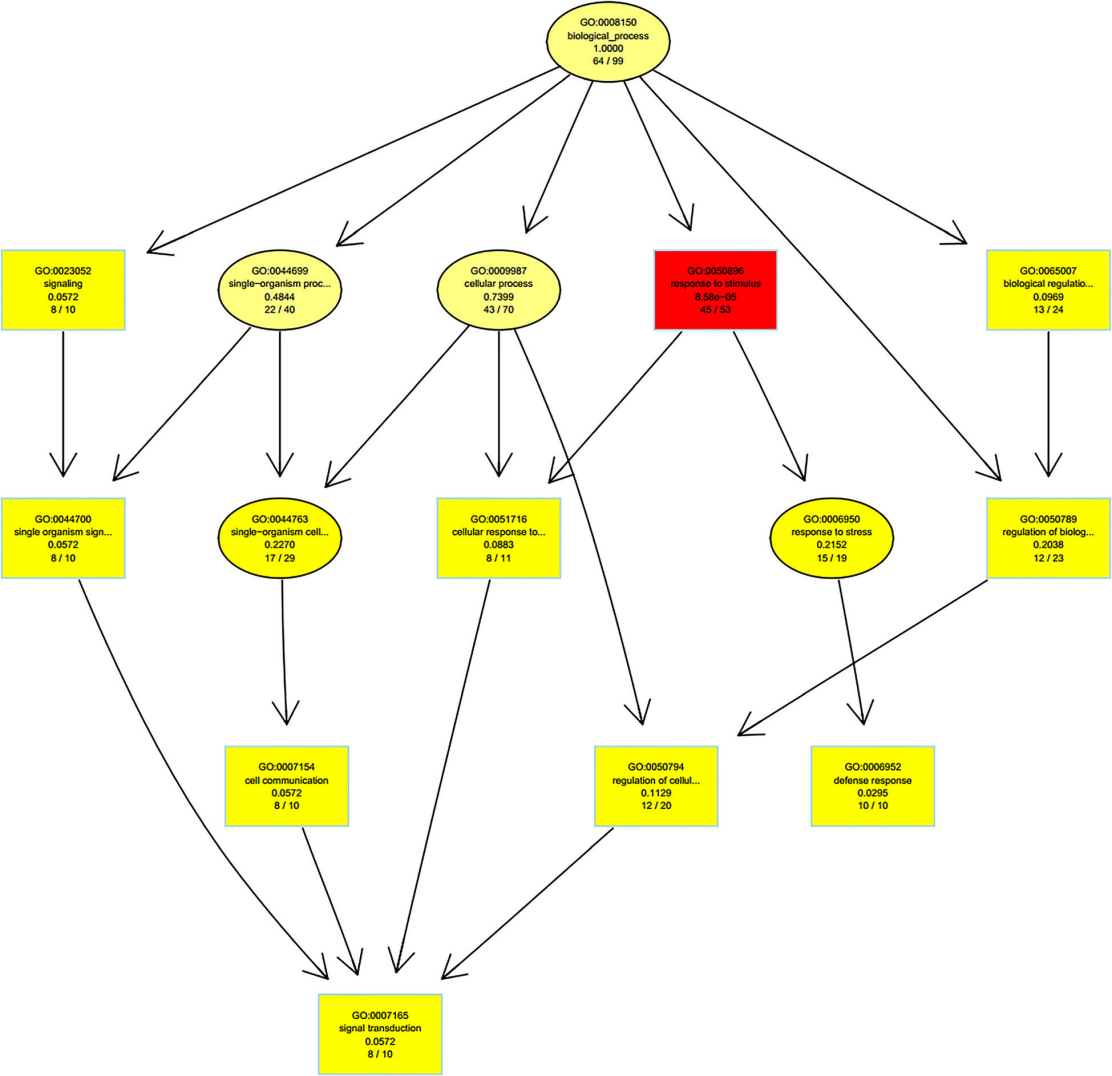
TopGO-directed acyclic graph for the target genes of differentially expressed miRNAs. Notes: enrichment was performed for each GO node, and the most significant 10 nodes are represented with boxes in the plot. The corresponding relationships between the layers are also included. The content description and enrichment significance value of the GO node is provided inside each box (or oval). Different colours represent different enrichment significance, and a deeper colour indicates a higher significance

The topGO software was used to perform enrichment analysis for the differentially expressed genes between the samples, and the hierarchical relationship of the significantly enriched terms in the GO system was intuitively displayed in the form of directed acyclic graph visualization. Biological process hierarchy analysis in GO enrichment for the target genes of the differentially expressed miRNAs between samples S01 and S02 is presented in Fig. 5. As noted in Fig. 5, a total of 76 enriched GO terms in the biological process classification was obtained. The most significantly enriched term is the response to stimulus of endogenous hormones and organic matter, followed by the regulation of metabolic processes of cell macromolecules (including biosynthesis of RNA, nucleic acids, and organic matter) and the regulation of gene expression. Of note, these terms enriched in different categories are enriched in cellular signal transduction. Cells sense the stimulation of information molecules through the cell membrane or intracellular receptors; thus, functions of the entire cell can be impacted. The transcriptional activity of target genes may be altered, thereby inducing the cellular response process. In the classification of Molecular Function, 38 enriched GO terms were identified. The most significantly enriched target genes included nucleotide binding followed by the activity of transferase, DNA, ribonucleosides, and ion binding. The above consensus results indicate that some *Larix olgensis* miRNAs are likely involved in regulation at the transcriptional level. The top 10 of topGO enrichment are the target genes in the classification of Molecular Function, as shown in Table 5.

**Table 5:** Statistics of topGO enrichment top 8

GO ID	Term	Annotated gene	Significant gene	Expected gene	KS
GO:0000166	nucleotide binding	73	62	49.07	1.10E-05
GO:0016740	transferase activity	37	29	24.87	0.01
GO:0016772	transferase activity, transferring phosp...	31	25	20.84	0.015
GO:0043531	ADP binding	11	11	7.39	0.04
GO:0043168	anion binding	53	42	35.62	0.049
GO:0032549	ribonucleoside binding	52	41	34.95	0.069
GO:0032559	adenyl ribonucleotide binding	52	41	34.95	0.069
GO:0017076	purine nucleotide binding	52	41	34.95	0.069

Notes: *Term* GO functional description, *Annotated gene* The number of genes annotated with this function among all the genes, *Significant gene* The number of target genes annotated with this function among the target genes of differentially expressed miRNAs, *Expected gene* The expected number of target genes with differentially expressed miRNA target genes, *KS* Statistical significance of enrichment terms, with a smaller KS value indicating more significant enrichment

As noted in the Table 5, the number of target genes of the differentially expressed miRNAs that were annotated as nucleotide binding accounted for 84.93% of the total number of target genes, which had the highest enrichment rate. Transferase activity, accounting for 78.38% of the total target genes that were annotated, exhibited the second highest enrichment rate. The enrichment of target genes for these two types of differentially expressed miRNAs is extremely significant, indicating that these miRNAs play an important role in life activities.

#### KEGG annotation and pathway enrichment in differential expression miRNA targets

In vivo, different genes interact with each other to perform biological functions. To further understand the functions of difference expression miRNAs and their targets, GO enrichment and KEGG pathway annotation analysis for all miRNAs and their targets were performed. The pathway significant enrichment analysis can help us ensure that differentially expressed target genes are involved in the main biochemical metabolic pathways and signal transduction pathways. The miRNA differential target genes of two samples were classified according to metabolic pathways, and two samples gave information of 16 metabolic pathways (Table 6). There are 8 miRNA target genes involved in the metabolic process, including valine, leucine and isoleucine biosynthesis, fatty acid biosynthesis, N-glycan biosynthesis, pantothenate and coA biosynthesis, butanoate metabolism, propanoate metabolism, pyruvate metabolism, and C5-Branched dibasic acid metabolism, which composed 50% of the differentially expressed target genes. In addition, enrichment target genes involved in the genetic information process were 5 terms that occupied 31.25%. In addition, there were plant hormone signal transduction and plant-pathogen interaction genes. This indicates that more than one metabolic pathway participates in combination during growth and development, and these metabolic pathways are completed coordinatedly by more than one miRNA. However, how the miRNAs regulate target genes needs further study and verification.

**Table 6:** Statistics of KEGG enrichment top 16

Kegg_pathway	ko_ID	enrichment_factor	Cluter_frequency	Genome_frequency	Regluate	*P*-value	Corrected_*P*-value	Target ID
Ribosome	ko03010	0.32	1 out of 8 (12.5%)	1 out of 25 (4%)	up (1)	0.32	1	c65500.graph_c0
Fatty acid biosynthesis	ko00061	0.32	1 out of 8 (12.5%)	1 out of 25 (4%)	down (2)	0.32	1	c82138.graph_c0
N-Glycan biosynthesis	ko00510	0.32	1 out of 8 (12.5%)	1 out of 25 (4%)	up (2)	0.32	1	c75325.graph_c0
Plant hormone signal transduction	ko04075	0.32	1 out of 8 (12.5%)	1 out of 25 (4%)	down (1)	0.32	1	c80883.graph_c0
Propanoate metabolism	ko00640	0.32	1 out of 8 (12.5%)	1 out of 25 (4%)	down (1)	0.32	1	c82138.graph_c0
Endocytosis	ko04144	0.32	1 out of 8 (12.5%)	1 out of 25 (4%)	up (1)	0.32	1	c79817.graph_c0
Butanoate metabolism	ko00650	0.32	1 out of 8 (12.5%)	1 out of 25 (4%)	up (2)	0.32	1	c83046.graph_c0
Valine, leucine and isoleucine biosynthesis	ko00290	0.32	1 out of 8 (12.5%)	1 out of 25 (4%)	up (3)	0.32	1	c83046.graph_c0
Ribosome biogenesis in eukaryotes	ko03008	0.32	1 out of 8 (12.5%)	1 out of 25 (4%)	down (1)	0.32	1	c40049.graph_c0
Plant-pathogen interaction	ko04626	0.32	1 out of 8 (12.5%)	1 out of 25 (4%)	down (1)	0.32	1	c71353.graph_c0
Pyruvate metabolism	ko00620	0.32	1 out of 8 (12.5%)	1 out of 25 (4%)	down (1)	0.32	1	c82138.graph_c0
C5-Branched dibasic acid metabolism	ko00660	0.32	1 out of 8 (12.5%)	1 out of 25 (4%)	up (1)	0.32	1	c83046.graph_c0
Protein processing in endoplasmic reticulum	ko04141	0.64	1 out of 8 (12.5%)	2 out of 25 (8%)	up (2)	0.546666667	1	c79817.graph_c0
Pantothenate and CoA biosynthesis	ko00770	0.64	1 out of 8 (12.5%)	2 out of 25 (8%)	up (1)	0.546666667	1	c83046.graph_c0
Spliceosome	ko03040	0.64	1 out of 8 (12.5%)	2 out of 25 (8%)	up (1)	0.546666667	1	c79817.graph_c0
RNA transport	ko03013	0.96	1 out of 8 (12.5%)	3 out of 25 (12%)	down (4)	0.704347826	1	c40049.graph_c0

Notes:*ko_ID* KEGG pathway ID, *Cluter_frequency* The ratio that sample-to-sample miRNAs predicted to target genes annotated to the pathway target genes number accounted for sample-to-sample predicted target genes of miRNAs annotation to KEGG. Genome_frequency: the ratio that target genes number of all miRNAs predicted were annotated to the target genes number of the pathway, which accounted for the target genes of all miRNAs annotated to the pathway; *P*-value: Significance of enrichment, the smaller the *P*-value, the higher the enrichment

#### Analysis of miRNA temporal and spatial expression patterns

Small RNA sequencing predicted 78 miRNAs. As *Larix olgensis* does not have a genomic database, the functions of the target genes of these miRNAs cannot be accurately defined. Based on the above research findings [33–40], eight miRNAs were chosen that may be related to growth and development to undergo additional quantitative fluorescence analysis to understand the relative expression of these miRNAs during several growth and development stages in larch. MiRNAs and target genes annotation are presented in Table 7. In addition, 5.8S rRNA was selected as the control reference, with the primer sequence 5’ACGTCTGTCTGGGCGTCG 3′. The upstream primer was a universal primer provided by the TransGen Biotech (Beijing, China). The downstream primers are presented in Table 8.

**Table 7:** miRNAs and target genes annotation in *Larix olgensis*

miRNA ID	miRNA	Target gene ID	Target gene annotation	Reference species
conservative_c67876.graph_c0_42013	miR160	c79046.graph_c0	auxin response factor 16	*Pinus massoniana*
		c47433.graph_c0	putative auxin response factor 10/16/17	*Cycas rumphii*
		c67876.graph_c0	hypothetical protein CCACVL1_17746	*Corchorus capsularis*
conservative_c100220.graph_c0_21	miR164	c82138.graph_c0	Carboxyl transferase	*Macleaya cordata*
		c51789.graph_c0	cup-shaped cotyledon	*Picea abies*
		c100220.graph_c0	ABC transporter ATP-binding protein	Archaeoglobus veneficus
conservative_c81519.graph_c0_113869	miR166–1	c75192.graph_c0	class III HD-Zip protein	*Pinus taeda*s
		c81519.graph_c0	unknown	*Picea sitchensis*
conservative_c101915.graph_c0_183	miR166–2	c101915.graph_c0	DEAD/DEAH box helicase	Pedobacter sp. KBW06
conservative_c53169.graph_c0_14321	miR396–1	c53169.graph_c0; c76194.graph_c0; c84439.graph_c0;	No	
		c80217.graph_c0	hypothetical protein COLO4	*Corchorus olitorius*
conservative_c84439.graph_c0_145702	miR396–2	c76194.graph_c0; c84439.graph_c0	No	
		c85259.graph_c0	mannitol dehydrogenase	Glonium stellatum
		c75801.graph_c0	unnamed protein product	Arabidopsis halleri
conservative_c79262.graph_c0_97269	miR950–1	c72897.graph_c0; c83878.graph_c0; c79262.graph_c0;	No	
		c70968.graph_c0	unknown	Picea sitchensis
		c69006.graph_c0	putative TIR-NBS-LRR protein	*Pinus monticola*
unconservative_c72897.graph_c0_60887	miR950–2	c60781.graph_c0	unknown	Picea sitchensis
		c79262.graph_c0; c83878.graph_c0; c72897.graph_c0;	No	
		c83910.graph_c0	NB-ARC	*Pinus tabuliformis*
		c79134.graph_c0	putative TIR-NBS-LRR protein	Pinus monticola

Note:target genes annotation was based on other species in Nr, Swissprot, Pfam database. “No” indicated no alignment results

**Table 8:** miRNA mature sequence and primers

miRNA	miRNA sequence (5′-3′)	length (nt)	reverse primer sequence (5′-3′)
miR160	ugccuggcucccuguaugcca	21	TGCCTGGCTCCCTGTATGC
miR164	uggagaagcagggcacgugcg	21	ATGGAGAAGCAGGGCACGT
miR166–1	ucggaccaggcuucauucccc	21	TCGGACCAGGCTTCATTCC
miR166–2	aaacgcauuucguacggacuga	22	AAACGCATTTCGTACGGACTGA
miR396–1	ucccacagcuuucuugagcuu	21	TCCCACAGCTTTCTTGAGCTT
miR396–2	gaaagcuguggaagagcau	19	GGGAAAGCTGTGGAAGAGCAT
miR950–1	ucacaucugggccacgaugguu	22	TCACATCTGGGCCACGATG
miR950–2	ugacaucugggccacgaugguu	22	TGACATCTGGGCCACGATG

For the selected reference 5.8S rRNA, when the sample template concentration and volume are basically the same, the expression of the 5.8S rRNA gene of all samples was relatively stable; the Ct value difference between samples was less than 2 and conformed to the reference standard. The dissolution curves of real-time quantitative PCR were all single peak, indicating that the PCR product was more specific.

#### Expression analysis of miRNAs in different tissues

**Fig. 6:**
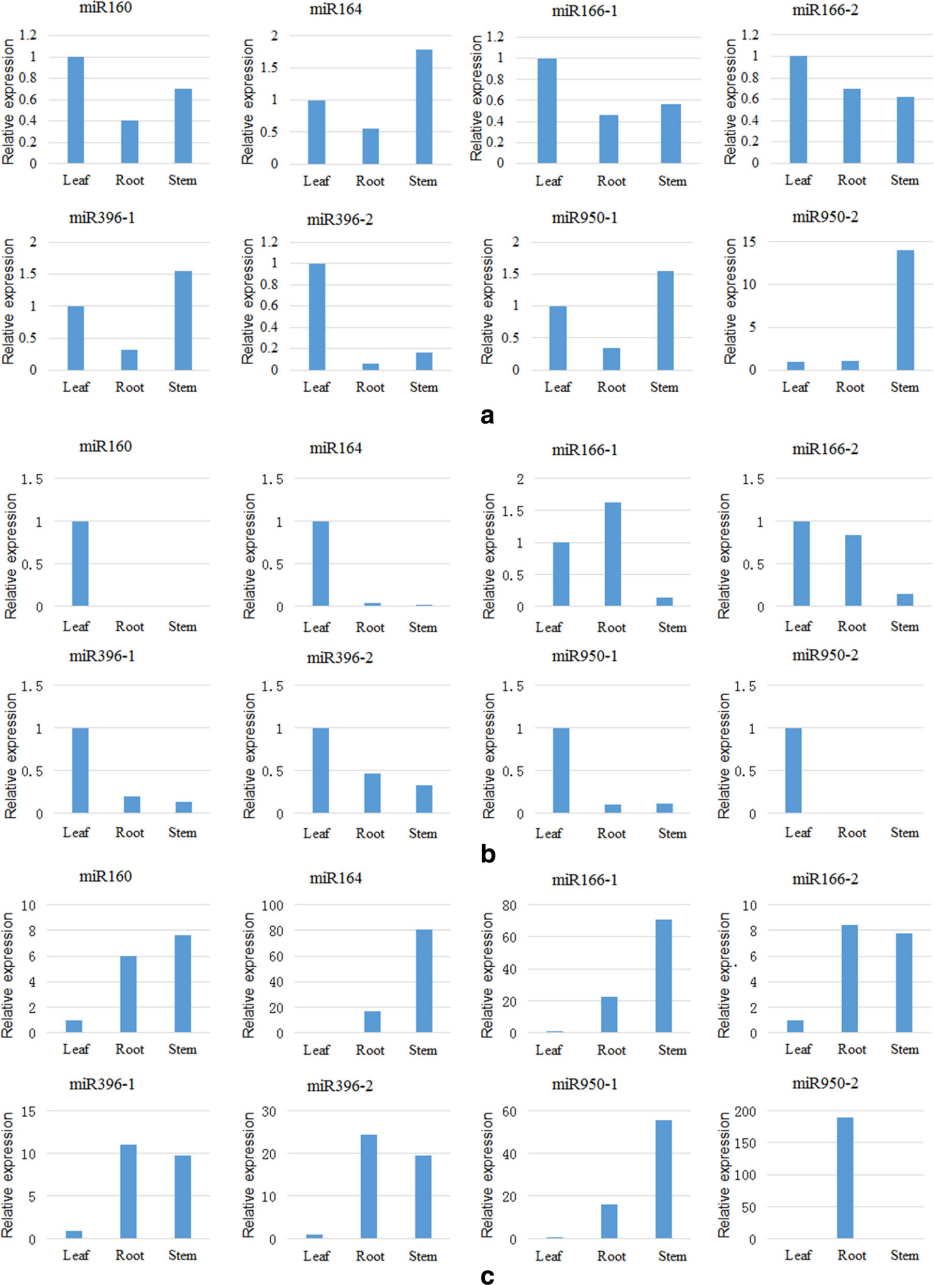
The relative expression of miRNAs in different tissues (roots, stems and leaves). Notes: **a**, **b**, **c** is miRNA relative expression at 60 days, 120 days, 180 days, respectively

Differences exist in the expression of the same miRNAs in different plant tissues. Using leaf tissue as the reference, the relative expression of each miRNA in the roots, stems and leaves of 60 days (120 days, 180 days) seedlings are presented in Fig. 6a (b, c).

As noted in Fig. 6, differences in miRNA expression in roots, stems, and leaves are noted at the same growth stage. At 60 days, miR160, miR166, and miR396–2 exhibited the highest expression in leaves; however, their expression in leaves was not significantly different from those in stems. miR950–2 exhibited the lowest expression in leaves, and its expression in stems was significantly increased compared with roots and leaves, indicating that miR950–2 likely played an important role in stem elongation. At 120 days, except for the slight increase in miR166–1 expression in roots, the relative expression of other miRNAs in the roots and stems decreased significantly. The highest expression was noted in leaves. In particular, the expression of miR160, miR164 and miR950–2 in stems was significantly reduced compared to leaves. These miRNAs were abundantly expressed in leaves potentially because they were closely related to the growth and development of leaves. At 180 days, miRNAs were abundantly expressed in roots and stems (miR950 expression in the roots was considerably increased compared to leaves), suggesting that these miRNAs are tightly connected with the formation of lateral roots and adventitious roots during this stage.

#### Expression analysis of miRNAs at different growth stages

**Fig. 7:**
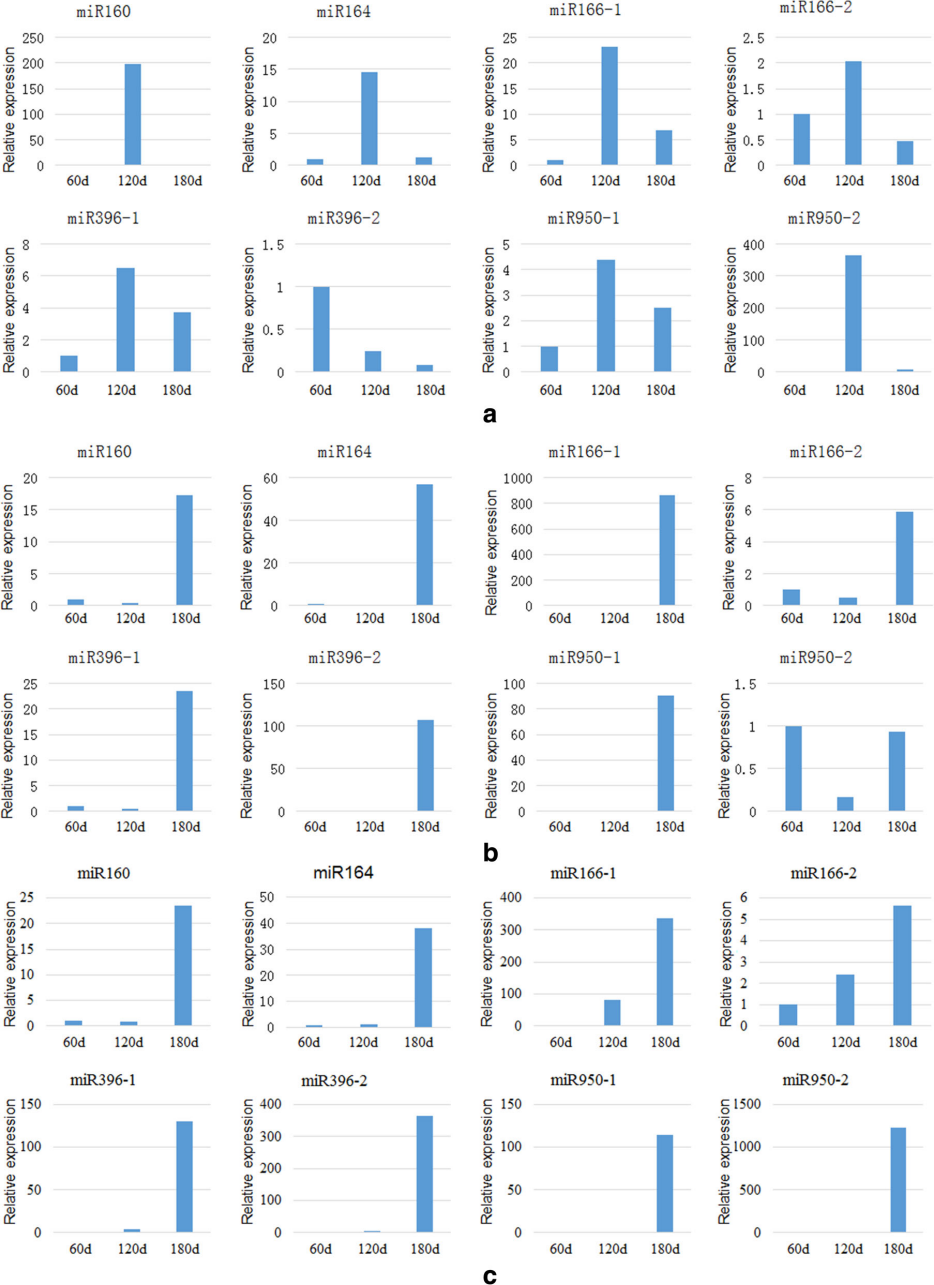
The relative expression of miRNAs at different periods (60 days, 120 days, 180 days). Notes: **a**, **b**, **c** is miRNA relative expression in leaf, stem, root, respectively

Understanding the differential expression and regulatory relationship of miRNAs and their target genes in roots, stems, and leaves at different growth stages aids in the exploration of the regulatory mechanisms and biological functions of miRNAs and their target genes in the growth and development of *Larix olgensis*. The relative expression of each miRNA in the root (stem and leaf) tissues at 60–180 days is presented in Fig. 7a (b, c).

As noted in Fig. 7, miRNAs exhibit different expression levels in roots, stems and leaves at different growth stages. miRNA has different variation trends among roots, stems and leaves. For the leaves, the relative expression of miR396–2 was reduced during the growth period, whereas the other seven miRNAs increased initially and then decreased. In particular, miR160, miR164, and miR950–2 exhibited a pronounced pattern of such a trend. On the other hand, the relative expression of miRNAs in the stems, miR166–1 and miR396–2, continuously increased, whereas the expression of all the other miRNAs, especially miR160, miR164, miR396–1 and miR950–1, first decreased and then increased. Similarly, the relative expression of miRNAs in roots, miR950–2 expression first decreased and then increased, whereas other miRNAs exhibited a trend of continuously increasing. Therefore, it was hypothesized that miRNA166–1 and miR396–2 expression in stems and roots increases with growth and development, indicating that they were likely related to the formation of xylem. In contrast, miR160, miR166–1, miR166–2, and miR396–2 exhibit the highest expression in leaves at the non-lignification growth stage but the lowest expression in the full lignification growth stage, indicating that these miRNAs may be involved in secondary metabolism in cells. Additionally, 60 and 120 days were the turning points for miRNA expression changes, indicating that they might be the key stages for miRNA to regulate the growth of *Larix olgensis*.

## Discussion

### sRNA sequencing methods

Small RNA sequencing analysis is a very important topic in miRNA research. This analysis involves the late-stage prediction and identification of miRNAs for further understanding the expression of miRNAs in specific tissues and at specific stages to screen for corresponding miRNAs in directed studies. Earlier studies isolated and identified miRNAs mainly via constructing libraries followed by direct cloning and sequencing. For example, Lu et al. [9] used this method to study the regulation of miRNAs in *Pinus taeda* after infection with the fusiform rust *Cronartium quercuum* f. sp. *fusiforme*. Yakovlev et al. [41] also used this method to study how small RNAs are involved in regulating the epigenetic mechanism of climate adaptation in zygotic embryos during the embryogenesis process in *Picea abies*. The method of library construction followed by direct cloning and sequencing is relatively simple and represents an effective and credible method for obtaining plant miRNAs. This method also allows the identification of known and new miRNAs. Nevertheless, such a method takes time, and the detection efficiency is low. In addition, the small RNAs obtained by this method include not only miRNAs but also other sRNAs, such as siRNAs, which severely hinders the identification of miRNAs. At present, the recent extensively adopted molecular biology experimental sequencing technologies make up for the above shortcomings. Since 2005, high-throughput sequencing technologies, such as massively parallel signature sequencing (MPSS) [42], 454-FLX [43] and Solexa, have been developed for the mining and analysis of small RNAs. Roussley et al. [44] performed high-throughput sequencing on wild rice using 454-FLX sequencing technology and found differentially expressed genes via alignment with their own rice genomes. Solexa is a novel sequencing method based on SBS (sequencing by synthesis), which utilizes a single-molecule array to perform PCR in a small flow cell. In addition, new reversible terminator technology is used to synthesize only one base at a time. Then, the fluorescent gene is labelled, and the fluorescence signal captured, which allows reading of the base information. Solexa sequencing generates reads within 35 bp in length, which are well suited for sequencing analysis of small RNAs, such as miRNAs. Additionally, millions or even billions of reads can be obtained in a short period of time. For conifers, miRNA studies can be performed through small RNA library construction and sequencing in *P. taeda* and *P. abies* [9, 10]. The characteristics of efficient and effective data collection using this method have largely accelerated miRNA research in animals and plants.

### Analysis of miRNAs and their targets

Previous studies indicated that many kinds of miRNAs were identified in angiosperms, and had been classified in the form of family [45, 46] but are rarely reported in the gymnosperms [47–49]. Recent studies have identified miRNA targets related to many aspects, mainly including transcription factors, signal transduction, plant growth and development [50], plant hormone signal transduction [51], RNA transporter [52], sRNA biosynthesis [53] and plant defence [54, 55]. However, for *Larix olgensis*, a study of miRNAs has not been reported previously. To obtain information pertaining to miRNA and target gene annotation, a sRNA library was constructed and then sequenced. Seventy-eight miRNAs and 333 target genes were obtained from 7,715,641 unique sequences, indicating that the growth and development process of *Larix olgensis* is regulated by many miRNAs. In addition, a large part of the sRNA was not annotated in this study, including sample S01 12,229,003 (79.96%) and sample S02 9,921,086 (83.27%), which may have been because there is no genome information in any of the databases for *Larix leptolepis*. Therefore, it is possible that there is also a large number of function-specific and novel miRNAs, suggesting that much information is available to be excavated.

When studying miRNA function, it is important to directly find the corresponding regulated target genes. The main target genes annotated were transcription factors, signal transduction, and cell wall and cell membrane biosynthesis in this study. In a previous study, the miRNA target genes were mostly transcription factors, which implies that miRNAs may participate in the regulation of more than growth and development. This study also suggested that a miRNA may regulate more than one target gene, and different miRNAs may also regulate the same target genes [56]. From the miRNA and target gene annotation information, it can be seen that miRNA has more than one target gene in *Larix olgensis*, and the same target gene not only regulates one miRNA. In addtion, miR950 was just annotated in conifer species, besides, there are functions unknown in some species, it suggested that miR950 may be special in *Larix olgensis.* Previous studies found that miRNAs were mainly negative regulation target genes. Theoretically, they should be negatively correlated, but there was very little negative correlation between the expression of miRNA and the target gene in maize. Therefore, it is possible that the regulation of target genes is affected by many factors, not just by miRNA regulation. In a pathway analysis study, the differentially expressed miRNAs were mainly involved in the biosynthesis of organic and macromolecules and inorganic ions and signal transduction, and target genes were not always involved in a metabolic pathway, For example, the predicted target genes of miR164 participate in three metabolic pathways and miR2592 in four metabolic pathways. It is also further confirmed that the interaction between miRNAs and their target genes is influenced by a variety of factors.

### Expression pattern of miRNAs by qRT-PCR

The temporal and spatial expression patterns of miRNA provide some ideas for functional research, which is also one of the important components of miRNA research. Different miRNAs are temporal- and tissue-specific in plants, showing that temporal and spatial expression may not only determine the functional specificity of tissues and cells but are also involved in complex gene regulation, which plays an important role in the growth and development of plants. Most of the miRNAs were differentially regulated in different tissues in *Prunus persica* [57]. In previous studies, miR164 could regulate floral organ development by cleaving CUC1 (cup 1 shaped cotyledon) and CUC2 mRNAs [33]. Moreover, the mechanism by which miRNAs regulate the translational mechanism of mRNAs is reversible, during which miRNAs and mRNAs are both expressed [40]. Cleavage of NAC1 (N-acetyl-L-cysteine) mRNA downregulates the hormone signalling of lateral root development [34]. Some *Prunus mume* NACs were downregulated during flower bud opening, and some NAC genes were highly expressed in stems during winter [58]. Auxin plays an important role in plant growth and development. ARF17, a member of the ARF (auxin response factor) gene family, was one of the target genes of miR160. miR160 negatively regulates ARF17 expression and plays an important role in response to early auxin signalling [35]. Overexpressing the precursor of ptcmiR396c under the control of pCaMV35S in the transgenic tobacco lines, the different lines showed delays in the growth and development and consequently delays in floral transition at three months stages of growth. Therefore, miR396 may play an important role in the growth and development of plants. [59]. In maize, miR166 regulated the differentiation of stem cells and miR166 enhanced plant adaptability to low-temperature stress by inhibiting NAC1 and ATHB-8 (*Arabidopsis thaliana* homeobox 8). Moreover, miR166c/f in the miR166 family may be related to the differentiation of plant microtubule cells and the formation of meristems and lateral roots; it is mainly involved in the metabolic process of auxin signal transduction, thereby regulating plant adaptability to stress conditions such that plants can develop towards favourable conditions [45]. MiR951 cleaves non-protein coding sequences homologous to plant disease-resistant proteins in *Pinus taeda* and produces short peptides or secondary siRNAs. It is hypothesized that miR950 may also exhibit this function [3]. Peptides are a class of plant signalling molecules that play important roles in growth and development and cannot be replaced by auxins or cytokinins [46, 47]. In *Brassica rapa*, many novel miRNAs, many with few target genes and low expression, suggest rapid evolution of the miRNA genes [60].

MiRNA is an important regulation gene that responds to environmental change rapidly. This study used qRT-PCR to detect the relative expression levels in leaves, stems and roots from three important periods in *Larix olgensis* growth and development. It was found that miR160, miR166 and miR396–2 had the highest expression in leaves at 60 days, but the expression of miR950 was the lowest, while in stems and leaves, it was significantly higher than that in roots. It is possible that the former is closely related to the growth and development of leaves, and miR950 may be related to the formation of the lateral root and lignin. At 120 days, it was just lignification. Except for miR166–1, other miRNAs had the highest expression in leaves. During the study period, the photosynthesis absorption of plants was relatively strong, so it was speculated that these miRNAs could promote the growth and development of leaves. When the plant was fully lignified over 180 days, the expression of all the miRNAs in the leaf was the lowest, but they were expressed abundantly in the stems. In particular, the expression of miR950 in roots was significantly higher than that in the stem, indicating that it plays a very important role in lateral root development. Previous studies had also found that miR950 can promote lateral root development in *Larix leptolepis*. The relative expression of miRNAs in roots, stems and leaves at different growth and development stages was found to be different, and the miRNA had different trends of variation. The relative expression of miRNAs first increased and then decreased in leaves, except miR396–2, while the result was a different expression pattern from stems and leaves, there was an increased trend in roots, except for miR950. It is possible that many miRNAs participate in the same metabolic process and play a very important role in the growth process. Identifying the related miRNAs would lay the foundation for growth and development mechanisms in *Larix olgensis*.

In previous studies, it was mentioned that the miR164 family regulated the R2R3-MYB structural protein involved in growth and development, signal transduction, and anti-stress response [61]. This indicates that miR164 plays an important role in growth and development as well as stress tolerance. The target gene NAC of miR164 can convert the plant hormone signal, consequently producing the lateral root in maize, and the increased expression of miR164 could reduce the lateral root [62]. This is consistent with the results of this study. When the plant grew for 120 days, the most vigorous development should have occurred in the lateral roots. At this time, the results show that the relative expression of miR164 was very low, which led to a large number of lateral roots. MiR166/miR165 played an important role in leaf curl, and it could inhibit the expression of the leaf roll gene rld by increasing miRNA expression and improving plant tolerance. In addition, miR166 was found to be involved in the regulation of root morphology in alfalfa. The expression of precursor miR166 reduced the expression of the class-III HD-ZIP (homeodomain-leucine zipper) gene, and there was abnormal development on the side of the symbiotic joint and lateral root [63]. In addition, miR160 also had a certain regulatory effect on leaf formation, indicating that the regulation of the leaf involves the participation of multiple genes. In this study, the expression of miR166 in the root increased as time went on, but increased first and then decreased in the leaf. Studies have also found that the target genes of miR160 and miR167 might play an opposite role in regulating the expression of GH3.6 (Gretchen Hagen3.6) [64, 65]; that is, the reduction of the miR160 target gene can activate the increase in auxin and adventitious roots, while miR167 target genes are the opposite. This also suggested that the miRNA was involved in the regulation of many levels of growth and development. This study predicted that the miR160 target gene was ARF. The lowest abundance expression of miR160 was found in root, but the expression of the target gene ARF was the highest in the analysis of the mature miRNA and target genes, speculating that it is possible to promote miR160 plant growth by regulating target gene ARF. Studies of miR396 showed that the target gene ARF is a transcription factor gene related to drought. However, this study discovered that miR396 plays a regulatory role in *Larix olgensis* growth and development, indicating that the gene had more than one function. MiR950 in the root was highly expressed at 180 days. As we all know, it is difficult to make *Larix olgensis* root in tissue culture by organogenesis or somatic embryogenesis. We can clone miR950, and then proceed to genetic transformation in *Larix olgensis,* thus improved root rate. In the future, the pathway of differentially expressed miRNA and their targets can be analysed to understand other functions or a regulation network of *Larix olgensis*.

## Conclusions

This study of 78 novel miRNAs has enriched the *Larix olgensis* genome. Differentially expressed miRNAs were analysed. Most of the expression levels were different in different tissues and stages, which suggested that miRNA expression levels are spatio-temporally variable. In short, the plant miRNA involved in every process of plant growth and development through the interaction between miRNA and its target gene can directly regulate plant growth and development and can also indirectly regulate growth and development of plants through their corresponding target genes. Some plant miRNAs physically clustered together as a polycistron, suggesting that these miRNAs have a function potentially regulating gene expression in a coordinated fashion. miRNAs in plants have important biological functions in the growth and development process, and studying their molecular mechanism has very important biological significance for regulating target genes in plant life activities. According to the expression pattern of miRNAs, it is possible to predict their functions. If validation of miRNAs and target gene function is needed, it is necessary to realize it through the genetic transformation of miRNAs. This is also a key problem urgently requiring a solution.

## Materials and methods

### Materials

#### sRNA sequencing materials

The materials for sequencing were collected from the Mao’ershan Experimental Forest Farm of Northeast Forestry University, Heilongjiang, China. According to the growth phenophase of *Larix olgensis*, the cambium and its surrounding parenchyma tissues were obtained at the beginning of the thickening growth period (May 5th) and the peak of the thickening growth period (June 15th), respectively. Samples were immediately placed in liquid nitrogen for cryopreservation and then taken to the laboratory and placed in a − 80 °C freezer until RNA extraction.

#### The qRT-PCR materials

For seed sowing and nursery growth, *Larix olgensis* plants were placed and grown in a thermostatic seedling chamber at 24 ± 1 °C with a light duration of 16 h.d^− 1^ and light intensity of 35 μmol·m^-2·s-1^. When the plants had grown for 60 days (not lignified), 120 days (beginning of lignification), and 180 days (completely lignified), three tissues (roots, stems, and leaves) were sampled. The collected samples were rinsed with pure water, dried with absorbent paper, and then quickly placed in liquid nitrogen for miRNA extraction.

### Methods

#### Construction and sequencing of sRNA library

##### sRNA library construction

The purity, concentration and integrity of RNA samples were detected using a Nanodrop spectrophotometer (BioRad, Philadelphia, PA, USA), a Qubit 2 fluorometer (Invitrogen, Carlsbad, CA, USA) and an Agilent 2100 bioanalyzer (Agilent Technologies, Santa Clara, CA, USA), respectively, to ensure that qualified samples were sequenced. For Illumina sequencing (Illumina, San Diego, CA, USA)), a sRNA library was constructed using the small RNA Sample Pre Kit as well as the sRNA passing quality test. Briefly, the small RNA (18–30 nt) was first connected to a 3′ adaptor; primers for the reverse reaction were added; and a 5′ adaptor was ligated to the small RNA. Finally, a reverse transcription reaction was followed by several cycles of PCR to obtain sufficient product for sequencing, and the target fragments were screened by PAGE gel electrophoresis. The small RNA library was constructed using all sequences from the fragments..

##### Library quality control and sequencing

After the sRNA library was constructed, a Qubit 2.0 fluorometer and an Agilent 2100 bioanalyzer were used to test the library concentration and insert size of the sRNA library, respectively. In addition, the Q-PCR method was used to accurately quantitate the effective concentration of the library to ensure the quality of the library. After accurate quantification, the sRNA was sequenced using the high-throughput Illumina sequencing platform HiSeq2500, and the sequence reads were single-end 50 nt products.

#### Statistical analysis of sequencing data

##### Production of clean reads

The raw reads obtained by sequencing contain an adaptor sequence or a low quality sequence. To ensure the accuracy of information analysis, it was necessary to control the quality of raw data and obtain clean reads. The quality control standards of the original sequence were as follows:

1. Filtering out the low quality reads (the base quality value is less than 30, which is occupied more than 20%);
2. Filtering out the reads with unknown base N whose content was more than 10%;
3. Cutting the 3′ end adapters and barcode sequence;
4. Discarding the reads smaller than 18 nt and longer than 30 nt.

##### The prediction of miRNAs and target genes

The miRNA transcription start site (TSS) is located in the reverse complementary sequence derived from the intergenic region, the intron and the encoding sequence, forming a precursor with a hairpin structure. The 60–70 nt precursor is transported to the cytoplasm and cleaved by the Dicer enzyme to form mature miRNA [66, 67]. The clean reads were mapped to the reference sequence (The reference here is based on based on transcriptome assembly sequence) using the program miRDeep2 to identify conserved and novel miRNA. Then, 18–30 nt nucleotide sequence alignment was undertaken for specific species in the miRBase database by miRDeep2. Sequences were unmapped to the reference sequence filtered by the eliminating adaptor, followed by extension of the miRNA base number to predict the miRNA structure and obtain the new miRNA. Finally, using the software TargetFinder to predict the target gene, the predicted miRNA of the highest sequencing abundance was used as the query sequence, and transcriptional data were used as a target gene screening database.

#### MiRNA extraction and reverse transcription

MiRNA extraction was performed using the EasyPure® miRNA Kit, a miRNA extraction kit purchased from TransGen Biotech (Beijing, China). The reverse transcription reaction of the miRNA was performed using TransScript® miRNA First-Strand cDNA Synthesis SuperMix (containing tailing enzyme and reverse transcriptase), a reverse transcription kit purchased from TransGen Biotech (Beijing, China). The cDNA obtained from reverse transcription was diluted 5-fold for use in qRT-PCR.

#### qRT-PCR analysis of miRNAs

Analysis results revealed that differentially expressed miRNAs were identified in *Larix olgensis*. To infer whether these miRNAs with either upregulated or downregulated expression exhibit tissue specificity or are involved in growth and development, the expression levels of some differentially expressed miRNAs in different tissues of *Larix olgensis* were analysed by qRT-PCR.

The qRT-PCR reaction for miRNAs was performed using a qRT-PCR kit from TransGen Biotech (Beijing, China), and SYBR dye was used in the reaction. The qRT-PCR instrument was ABI7500 (Applied Biosystems, USA, and PCR instrument used for gene expression analysis). Three technical replicates were included for each reaction. The reaction procedure was as follows: 94 °C for 30 s; 40 cycles of 94 °C for 5 s, 60 °C for 15 s, 72 °C for 35 s; 95 °C for 15 s; 60 °C 1 min; 95 °C 30 s. When the amplification reaction was completed, melting curve analysis was performed to ensure that the melting curve was unimodal, to ensure the unity of the PCR amplification product, which also indicated that the PCR amplification product exhibited high specificity.

Because plant cells contain a cell wall and are more difficult to lyse, the cell tissue is made grinding powder using liquid nitrogen grinding. The spoon, mortar and pestle were placed in the autoclave at 121 °C for 15 min. Then, 0.1% DEPC water was used to treat the plastic materials in order to prevent the RNA enzyme from degrading RNA. In addition, in the last step of the experiment, when adding RNase free H_2_O, it is best to place it into a constant-temperature water bath to preheat to 60 °C and add it to the centre of the filter as soon as possible, which can improve the extraction efficiency of miRNA. Before the real-time quantitative PCR, the added MIX must be fully mixed in order to reduce the effect on the error of the addition.

#### Data collation and statistical analysis

The raw data from this study were processed with Excel 97–2003. The relative expression of miRNAs in each treatment was quantitatively analysed using the 2 ^-ΔΔCt^ method [68]. Using 5.8S rRNA as the internal control gene for mature miRNAs, the gene expression levels of miRNAs were normalized. Calculation and analysis of miRNA differential expression were subsequently performed.

## Data Availability

The raw sequences generated during the current study are available in the National Center for Biotechnology Small Reads Archive (NCBI SRA, http://www.ncbi.nlm.nih.gov/sra) under Bioproject PRJNA544580 and BioSample accessions SAMN11843655, SAMN11843656. The datasets generated or analyzed during this study are included in this published article.

## Abbreviations

AGO1: Argonaute1
ARF: Auxin response factor
ATHB-8: *Arabidopsis thaliana* homeobox8
COG: Clusters of Orthologous Groups of proteins
CUC1: Cup 1 shaped cotyledon
DCL: Dicer-like
FC: Fold change
FDR: False discovery rate
GH3.6: (Gretchen Hagen3.6)
GO: Gene Ontology
HD-ZIP: Homeodomain-leucine zipper
KEGG: Kyoto Encyclopedia of Genes and Genomes
miRNA: MicroRNA
MPSS: Massively parallel signature sequencing
NAC: N-acetyl-L-cysteine
nt: Nucleic acid
qRT-PCR: Quantitative real-time PCR
RISC: RNA-induced silencing complex
SBS: Sequencing by synthesis
SE: Somatic embryogenesis
sRNA: Small RNA
TPM: Transcripts per million
TSS: Transcription start site
